# Latency: the hidden HIV-1 challenge

**DOI:** 10.1186/1742-4690-3-7

**Published:** 2006-01-16

**Authors:** Alessandro Marcello

**Affiliations:** 1 Laboratory of Molecular Virology, International Centre for Genetic Engineering and Biotechnology (ICGEB), Padriciano, 99 – 34012 Trieste, Italy

## Abstract

Eradication of HIV-1 from an infected individual cannot be achieved by current regimens. Viral reservoirs established early during the infection remain unaffected by anti-retroviral therapy for a long time and are able to replenish systemic infection upon interruption of the treatment. Therapeutic targeting of viral latency will require a better understanding of the basic mechanisms underlying the establishment and long-term maintenance of HIV-1 in resting memory CD4 T cells, the most prominent reservoir of transcriptionally silent provirus. Since the molecular mechanisms that permit long term transcriptional control of proviral gene expression in these cells are still obscure, this review aims at summarizing the various aspects of the problem that need to be considered. In particular, this review will focus the attention on the control of transcription imposed by chromatin through various epigenetic mechanisms. Exploring the molecular details of viral latency will provide new insights for eventual future therapeutics that aim at viral eradication.

## Introduction

The major obstacle to HIV-1 eradication is the establishment of a latent infection. In infected individuals, viral production is a dynamic process involving continuous rounds of infection of CD4+ T lymphocytes with rapid turnover of both free virus and virus-producing cells that have a half-life of 1–2 days [1,2]. The decay curves of plasma viremia following antiretroviral treatment have shown that after an initial fast decay, that wipes out the majority of circulating viruses in 1–2 weeks, plasma virus declines at a lower rate [3,4]. The half-life of this compartment was estimated to be 1–4 weeks, but the nature of the cellular reservoir responsible for the second phase in the decay curve is still unclear. These cells could be macrophages, which are less sensitive to the cytopathic effect of HIV-1 infection [5] and that once terminally differentiated have a turnover rate of approximately 2 weeks. In addition, cellular reservoirs for HIV-1 could also be CD4+ T lymphocytes not fully activated, which carry the integrated provirus in a non-replicative state until the activation process is complete. Finally, dendritic cells (DCs) may also delay the release of infectious virus, since they are not permissive for HIV infection but can carry the virus trapped on their surfaces [6].

After two months on HAART the plasma levels of genomic RNA falls below the limit of detection in most previously untreated patients. Therefore, it was initially assumed that prolonged treatment might lead to eradication of the virus in these patients [3]. Unfortunately, it is now clear that long-lived reservoirs of HIV-1 can persist for years in the presence of HAART. Although certain tissues like the male urogenital tract or the central nervous system might preserve infectious virus [7,8] the reservoir that appears to be the major barrier to eradication is composed of latently infected resting memory CD4+ T cells that carry an integrated provirus that is transcriptionally silent [9,10]. The extremely long half-life of these cells, combined with a tight control of HIV-1 expression, make this reservoir ideally suited to maintain hidden copies of the virus, which are in turn able to trigger a novel systemic infection upon discontinuation of therapy. Given the importance of this reservoir, a lot of effort has been invested to characterize these cells from infected patients. These studies will be discussed in the following chapters, which will also address the problem of choosing appropriate model systems to thoroughly characterize the molecular determinants that allow the provirus to remain silent. Such mechanisms are mostly related to transcriptional control of viral expression and they depend both on the host cells and the virus. Finally, some ideas on how to approach viral eradication in the light of these novel findings will be presented in the final chapter.

## Source of latently infected cells

HIV-1 exploits different strategies to persist within infected individuals. In CD4+ T lymphocytes, the replicative state of the virus is dependent upon the cell cycle of the host cell. Whereas HIV-1 entry into activated CD4+ lymphocytes leads to a productive infection [11], the virus encounter several blocks prior to integration in resting CD4+ lymphocytes [12]. Such post-entry blocks have been proposed to result from a delay in completing reverse transcription due to low nucleotide pools and to the inability to import the pre-integration complex into the nucleus [13-16]. Most recently the anti-retroviral deoxycytidine deaminase APOBEC3G has been shown to strongly protect unstimulated peripheral blood CD4+ T cells against HIV-1 infection [17]. Furthermore, in Old World primates, TRIM5α, a component of cytoplasmic bodies, confers a potent block to human immunodeficiency virus type 1 (HIV-1) infection that acts after virus entry into cells, probably at the level of capsid processing [18]. While these blocks delay the production of progeny virus following the infection of CD4+ resting T cells, mitogenic stimuli are able to trigger viral replication and release of infectious virus [13,14,19-21]. Although one would expect that activation of the cell per se would allow more efficient reverse transcription and nuclear import, by increasing the pools of DNA precursors and the available ATP used for the active mobilization of the PIC, still it is possible that specific blocks must be removed to allow full recovery of HIV-1 infectivity. In the case of APOBEC3G for example, activation of resting T cells induces the shift of the active low-molecular-mass form of APOBEC3G to an inactive high-molecular-mass complex unable to restrict viral infection [17]. Other types of replication blocks that act after provirus integration in resting T cells, like for example the inhibition of NF-κB activity by Murr1 [22], will be discussed in the following chapters.

Regardless of the kind of restriction imposed by the resting T cells on viral replication, this reservoir of pre-integrated latent virus is relatively labile persisting for weeks and cannot be accounted for the long-term latency observed during HAART.

In addition to CD4+ T lymphocytes, dendritic cells and macrophages are considered reservoirs for HIV-1 infection, but information on the replicative state of the virus within these cells is limited. DCs capture and internalize extracellular virions via the DC-SIGN lectin. Captured virions can subsequently be transmitted to T cells in trans [6]. However, DC-SIGN does not significantly protect captured virions against degradation, leading to loss of infectivity within several hours [23]. In HIV-1-infected monocyte-derived macrophages, mature viral particles can be observed within late endosomes [24]. Virions found within monocyte-derived macrophages persist and retain infectivity for weeks, thus providing an additional mechanism for viral persistence [25]. HIV-1 hidden in DCs and macrophages certainly plays an important role for viral spread and cell-cell transmission, but its involvement in long-term viral persistence has yet to be demonstrated.

A more stable form of latency occurs in CD4+ T cells that carry an integrated provirus. In principle, since integration requires T cell activation to allow efficient reverse-transcription and nuclear import of the pre-integration complex, post-integration latency can result only from the return of an infected activated T cell to a quiescent state. Evidence to this model has come from studies from Siliciano and co-workers who demonstrated the existence of resting memory CD4+ T cells carrying an integrated provirus in vivo [9]. The phenotype of these resting cells carrying a non-productive HIV-1 infection, derived from peripheral blood of patients undergoing antiretroviral therapy with low to undetectable viremia, indicates that they derive from infected CD4+ lymphoblasts that have reverted to a resting memory state. These cells show a specific set of surface markers (such as CD4+, CD25-, CD69-, HLA-DR-) and are positive for the integrated provirus. Most importantly, upon mitogen stimulation, infectious virus can be recovered from these cells, indeed demonstrating that they represent a true, inducible viral reservoir. Hence, *in vivo*, it appears that HIV-1 gets trapped in T cells that revert to a resting memory state. In some respect long term HIV-1 persistence reflects directly long-term T-cell memory. At any given time, most CD4+ T lymphocytes in the body are in a resting G0 state. In response to antigens, resting T cells undergo a burst of cellular proliferation and differentiation, giving rise to effector cells. Most effector cells die quickly, but a subset survives and reverts to a resting G0 state (contraction phase). These lymphocytes persist as memory cells, with an altered pattern of gene expression enabling long-term survival and rapid responses to the relevant antigen in the future. Activated CD4+ cells are highly susceptible to HIV-1 infection and typically die quickly as a result of the cytopathic effects either of the virus or of the host immune response. However, some activated CD4 cells may become infected and then survive long enough to revert back to a resting state. Unfortunately, our current understanding of the decisive factors that determine if a CD4+ T cells will die or become a memory cell, as well as those that allow the self-renewal of resting memory cells for a lifetime, is still largely incomplete.

## Models for latently infected cells

Despite the great wealth of information on the regulation of HIV-1 transcription, the crucial molecular events that control maintenance of the quiescent state in resting T cells remain elusive. Part of the problem depends on the lack of an appropriate model system. Most cell lines carrying an integrated quiescent provirus have been derived from transformed lymphoblasts that have been infected *ex vivo *with HIV-1. After an initial burst of viral replication and cell death, a population of non-productive cells carrying an integrated provirus remains. Establishment of latency in these cell lines is driven by selection of cells that resist viral replication and has been linked to mutation in viral genes, to certain cellular proteins and to the site of integration [26-29]. Alternatively, T cells can be transduced with an HIV-1 vector carrying Tat and a reporter gene. This method has allowed the characterization of the integration status irrespective of the replication of the virus and has provided useful insights on the status of the integrated provirus. However, the constantly activated and proliferating nature of these cells, either infected or transduced, does not accurately represent the quiescent cellular environment of latently infected cells in vivo. A convenient animal model that recapitulates HIV-1 latency does not exist. In fact, several blocks to HIV-1 infection in mice greatly impair the development of an animal model to study HIV-1 infection amenable to genetic manipulation. One possibility would be the use of the SCID-hu (Thy/Liv) mouse that carries a source of human hematopoietic progenitor cells and human fetal thymus to provide a microenvironment for human T cells lymphopoiesis. Latently HIV-1 infected CD4+ T cells can be obtained in this model, but the exact extent to which it can be applied to natural infection is not known [30]. SIV macaque models of AIDS are well established and have been extremely useful in HIV-1 vaccine development and in advancing the understanding of the pathogenesis of AIDS. The first report exploiting the SIV-macaque model to study viral infection in the course of antiretroviral therapy showed persistence of the virus in resting CD4+ T cells with many similarities to the human situation [31]. This study provides initial evidence for the utility of a closely related retroviral infection for the analysis of HIV-1 persistence during antiretroviral treatment. However, the complexity of the protocol, which also requires antiretroviral drugs especially designed to control SIV infection, makes this model impractical if only for the preclinical evaluation of novel strategies to target viral reservoirs.

## Possible molecular mechanisms behind latency

Since the HIV-1 provirus is found integrated into the host genome, regulation of viral gene expression depends on the chromatin environment at the site of integration and on the interaction of the viral Tat *trans*-activator with host factors. Clearly, multiple mechanisms could concur in this process.

### I) Cis- and trans-acting factors involved in HIV-1 silencing

The U3 region of the HIV-1 LTR functions as the viral promoter and contains consensus sequences for several transcription factors, including NFAT and NF-κB, involved also as positive regulators of cell activation in uninfected T cells [32]. NF-κB is a key host transcription factor required for LTR activation [33]. In resting T cells NF-κB is sequestered in the cytoplasm bound to IκB and it is transported to the nucleus following cellular activation by TCR engagement or stimulation by IL-2 or TNFα. NF-κB interacts with two highly conserved binding sites found in the viral LTR and promotes transcriptional activation. Murr1, previously known for its involvement in copper regulation, has been recently shown to inhibit basal and cytokine-stimulated NF-κB [22]. Most importantly, knockdown of Murr1 by RNAi in primary resting CD4+ lymphocytes increased HIV-1 replication. Thus, Murr-1 acts as a host restriction factor that inhibits HIV-1 replication in resting T cells.

In addition to host transcription factors, HIV-1 transcription is boosted by the viral Tat *trans*-activator, a highly unusual protein that interacts with a *cis-*acting RNA element (trans-activation-responsive region; TAR) present at the 5' end of each viral transcript [34]. Through this interaction, the protein activates HIV-1 transcription by promoting the assembly of transcriptionally active complexes at the LTR through multiple protein-RNA and protein-protein interactions. Tat interacts directly with cyclin T1, the cyclin component of CDK9, which phosphorylates the carboxy-terminal domain of RNA polymerase II to enhance its processivity [35,36] (reviewed in: [37]). Tat-induced transcriptional activation of the LTR promoter is concomitant with recruitment of the transcriptional co-activators p300 and the highly homologue cAMP-responsive transcription factor binding protein (CBP) [38-41]. These large proteins are histone acetyl-transferases capable of modulating the interaction of nucleosomes with DNA and with other factors involved in transcription. In fact, besides histones, Tat itself is a substrate for the enzymatic activity of p300/CBP and of the associated factor P/CAF and is regulated by acetylation/deacetylation [42-47]. Furthermore, Tat is also tightly regulated by ubiquitination, further highlighting the intimate interplay between the viral trans-activator and the host cell [48].

Tat-associated proteins could be one of the limiting factors for processive transcription in resting T cells. In this respect, the low levels of P-TEFb kinase activity (CDK9 and Cyclin T1) that have been observed in resting T cells are increased in response to activating stimuli [49]. Tat itself could be the main limiting factor being subject to tight post-translational regulation by acetylation and ubiquitylation [42,46,48]. These data fit in a model where limiting availability of host cell's factors and/or the viral *trans*-activator concur in maintaining the virus transcriptionally silent. Possible mechanisms propose premature termination of transcription due to the absence of sufficient concentrations of Tat and NF-κB [50,51] or inefficient export of RNAs for structural proteins [52]. A recent report has also shown that fluctuations in Tat expression alone govern stochastic gene expression of the viral LTR [53]. However, when analyzing these studies one should always keep in mind that they should hold true also in resting T cells *in vivo.*

### II) Integration-site-dependent determinants of HIV-1 silencing

HIV-1 is found integrated into the genome of resting memory T cells, hence the chromatin status at the site of integration determines whether the provirus is transcriptionally active, poised for activation or inactive. A recent report [54] has analyzed the integration site of HIV-1 in resting memory CD4+ cells derived from patient on highly active antiretroviral treatment. Surprisingly, HIV-1 has been found in intronic regions of actively transcribed genes. Consistently, HIV-1 sequences were included in the unspliced RNAs of these genes. These findings, although complicated by the high levels of dead integration events observed (i.e. only a small fraction of resting T cells carrying a silent provirus becomes productive when activated), correlate with the observation that HIV-1 integrates in transcriptionally active genes during productive infection of cultured T cells [55], but are in sharp contrast with previous work showing that HIV-1 infected T cell lines selected for a quiescent state of the provirus show preferential integration into heterochromatin [28,56]. A very recent study helps clarifying this point showing that the integration site of quiescent/inducible HIV-1 vectors in T cell lines could be associated with heterochromatin, as reported, but also with actively transcribing genes, thus confirming the analysis in patients' cells [57]. Another interesting observation of this work is that the inducible state of the HIV-1 provirus could depend also upon integration in intergenic regions of gene-poor chromosomes. However, all these three conditions: integration into heterochromatin, integration into highly transcribed genes and integration into gene poor chromosomes, account for only 40% of the inducible integration events in this system. Notably, stochastic gene expression from the viral LTR, a phenotype dependent on the levels of Tat, occurs with a high frequency within 1 kb of a human endogenous retrovirus LTR [53]. These observations deserve further study since they may indicate that other as yet not identified chromatin environments at the site of provirus integration control transcriptional silencing and reactivation. In fact, a totally different pattern might emerge, particularly considering novel concepts on the relationship between spatial positioning of chromosomes within the nucleus and transcription activity [58]. T cells switching from a memory state to a lymphoblast and vice-versa undergo a program of spatial genome reorganization of specific genes [59-61]. HIV-1 proviruses "trapped" in a gene that is being spatially confined in a silenced state might become inactive and poised for activation from external stimuli [62].

So far we have considered post-integration transcriptional silencing as a passive consequence of chromatin status. However, we might also consider a certain degree of bias on the integration site driven by the association of the pre-integration complex with certain cellular factor such as the high mobility group (HMG) protein A1, the barrier to autointegration (BAF), the INI1 homologous of the SNF5 component of the ATP-dependent chromatin remodeling complex SWI/SNF and LEDGF/p75 [63-65]. BAF binds directly to double-stranded DNA, nuclear LEM-domain proteins, lamin A and transcriptional activators [66]. Recent observations suggest that BAF has structural roles in nuclear assembly and chromatin organization and might interlink chromatin structure, nuclear architecture and gene regulation. Integrase associates also with INI implying a role of transcription-related ATP-remodeling complexes in determining integration. Particularly interesting is the fact that INI associates with the promyelocytic leukemia protein PML, the principal component of the nuclear bodies [67]. These nuclear structures, whose function is still largely unknown, are dynamically associated to the transcriptional Cyclin T1 and to co-activators such as p300/CBP [68]. Intriguingly, IN binds also p300 and this interaction is involved in the integration process through acetylation of IN itself [69]. As a note of caution it should be said that none of the above mentioned factors that interact with the viral integrase has been shown to be functionally expressed in resting CD4+ T cells in relation to HIV infection. Future research will tell us more on these interactions of HIV-1 integrase and their role in HIV-1 integration.

### III) A role for RNA interference in HIV-1 silencing?

RNA Interference (RNAi) was first identified as a post-transcriptional response to exogenous double-stranded RNA (dsRNA) introduced in *C. elegans*, but this mechanism is conserved from plants to nematodes and mammals [70-72]. RNAi is triggered by long dsRNA cleaved by the cytoplasmic RNaselll enzyme Dicer into short, interfering RNAs (siRNAs). One strand of the siRNA is incorporated into the effector complex of RNAi, the RNA-induced Silencing Complex (RISC). The short RNA guides RISC to target complementary mRNA and catalyzes an endonucleolytic cleavage, resulting in post-transcriptional gene silencing (PTGS) of gene expression. In mammalian cells, siRNAs are recognized by the pathway responsible for the activities of a class of endogenous 21–22 nt microRNAs (miRNAs) (for a recent review see [73]). miRNAs are first produced as long hairpinned precursor dsRNAs transcribed by RNA polymerase II and are sequentially processed by the nucleases Drosha and Dicer. The short dsRNA produced are thought to regulate gene expression mainly at the translational level. Many different miRIMA genes have been predicted in humans, and they have been implicated in the regulation of genes involved in development and growth control [74].

RNAi-mediated pathways of transcriptional silencing have also been shown to induce chromatin modifications at the homologous genomic locus in plants and lower eukaryotes (for a review: [75]). Transposable elements and related repeats are primary targets for RNAi-mediated pathways in the nucleus, consistent with a role for RNAi in host defense against invasive viral sequences (for a recent review: [76]). Silencing occurs through CG methylation by specific DNA methyltransferases that are directed to the target by the methylation of lysine 9 of histone 3 (H3K9-Me). These findings have led to a model whereby siRNAs directed *de novo *DNA methylation through the successive action of a histone methyltransferase and DNA methyltransferases that maintain methylation at target DNA loci (RITS, RNA-induced transcriptional silencing) [77].

Artificial RNAi can efficiently suppress several human viruses, including HIV-1 [78,79]. However, this effect is shot-term, since the virus is capable of evading the siRNA response by random mutation of the target sequence, thus limiting the efficacy of this antiviral approach [80]. Not surprisingly, several viruses encode also their own miRNA that can modulate viral replication (reviewed in: [81]). Herpesviruses like EBV and HSHV encode miRNAs directed against cellular and viral targets and are believed to regulate the latent/lytic transition of these viruses [82,83]. Short-hairpin siRNA precursors have also been found in the HIV-1 genome. Such sequences are involved in suppression of viral transcription unless counteracted by the inhibition of endogenous Dicer activity targeted by the viral Tat transactivator [84]. This mechanism is similar to what has been observed for the primate foamy retrovirus PFV-1 that is capable of subverting a cellular miRNA block through the activity of the viral Tas protein [85]. Another HIV-1-encoded miRNA has been identified in the nef gene and has been speculated to be a possible determinant of long-term non-progression to AIDS through inhibition of Nef function [86].

It would be intriguing to speculate also about the existence of a transcriptional pathway of HIV-1 gene silencing, taking into account previous observations that might [87] link CpG methylation at the HIV-1 promoter to transcriptional silencing [88]. As a note of caution, however, it should be observed that at present RNAi mediated transcriptional gene silencing in human cells is highly controversial, and care should be taken in extrapolating data obtained in lower eukaryotes. Nevertheless, models such as HIV-1 could help in disclose these archival protective mechanisms in human cells, if they exist.

## Potential therapies to eliminate latently infected cells

Although the implementation of HAART has improved the survival and quality of life of HIV-infected individuals, HIV cannot yet be eradicated from infected individuals. Several studies have demonstrated that in individuals receiving HAART, the frequency of HIV-infected cells is reduced to fewer than one cell per 10^6 ^resting CD4+ T cells [10,89,90]. However, even after years with viremia below the limit of quantification, the frequency of these infected cells does not decrease further. The therapeutic approaches evaluated to date have failed to demonstrate a significant and persistent decline of this latent viral reservoir [91], which appears small but stable and contains both wild-type and drug-resistant viral species [92]. Several studies have shown that intensive antiretroviral therapy in combination with interleukin-2 or global T cell activators fails to eradicate HIV-1 infection. Global T cell activation may instead induce viral replication and increase the number of susceptible uninfected target cells beyond the threshold that can be contained by antiretroviral therapy [93]. Following this approach, a strategy that selectively activates quiescent proviral genomes with limited effects on the host cell exploited the properties of the phorbol ester Prostratin or the human cytokine interleukin-7 that have been reported to reactivate latent HIV-1 in the absence of cellular proliferation [30,94-96]. Another promising agent that has been proposed is the histone-deacetylase inhibitor Valproic acid capable of inducing outgrowth of HIV-1 from resting CD4+ cells of aviremic patients without full activation of the cells from the quiescent state [97]. Such treatments, in combination with antiretroviral therapy, should allow outgrowth of latent HIV-1 but avoid the pitfalls of global T cell activation.

Another approach would be to target and destroy CD4+ memory cells. A study has been conducted *ex vivo *with an anti-CD45RO ricin immunotoxin to decrease the number of latently infected CD4+ T cells obtained from HIV-infected individuals without detectable plasma viremia [98]. Such treatment significantly reduced the frequency of CD4+ memory cells with only a modest effect on the memory responses of CD8+ T cells. Therefore, purging latent cells from infected individuals on highly active antiretroviral therapy might reduce the HIV latent reservoir without seriously compromising CD8+ T cell memory responses. However, this kind of approach targets both infected and non-infected resting T cells and would severely deplete the immunological memory of the patient.

## Conclusion

**Figure 1:**
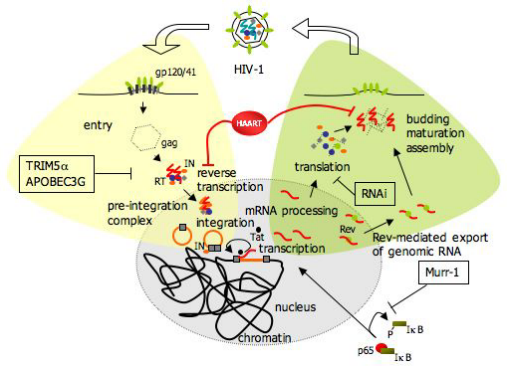
Schematic description of the HIV-1 life cycle highlighting the various blocks that can delay viral replication leading to prolonged hiding of the virus in the host cell during HAART. These include: (i) pre-integration blocks like the deoxycytidine deaminase APOBEC3G, the cytoplasmic body component TRIM5α (in Old World monkeys), incomplete reverse-transcription and defects in nuclear import; (ii) post-integration blocks such as integration into heterochromatin where transcription is repressed, ineffective RNAPII elongation in the absence of Tat or of key host factors, regulation of NF-kB by Murr-1; (iii) translational blocks induced by RNAi.

Recent advances have identified a long-lived stable reservoir of HIV-1 in patients on effective antiretroviral therapy that could potentially persist for life. This reservoir consists of a small pool of resting CD4+ T cells carrying an integrated provirus that somehow control viral expression allowing the virus to remain undetectable in plasma (Figure 1). Discontinuation of therapy and activation of cells results in production of infectious virus leading to a novel systemic infection. Hence, elimination of this reservoir by novel therapeutic approaches will be required before eradication can be achieved. Our knowledge of the cellular and molecular mechanisms behind the establishment of this remarkably stable reservoir will depend on appropriate in vitro model systems and in vivo animal models. These should allow the dissection of the pathways leading to transcriptional control of HIV-1 replication. In particular it will be crucial to correlate the activation state of host cells with the transcription state of the provirus. Key elements to be studied are: the site of provirus integration, which is determined by the viral integrase, and the mechanisms that control gene expression at these chromatin loci, which depend mostly on the activity of the Tat transactivator.

It is time to seriously consider alternative therapies that take into account the aim of eradicating infectious virus from the patients. In order to do so an effort is needed toward the understanding of the intimate interplay that is established at the molecular level between the virus and the host cell. From such studies it will be possible to devise novel therapies that selectively target the hidden virus.

## List of abbreviations

HIV-1, human immunodeficiency virus type 1; HAART, highly active antiretroviral therapy; CD4/CD3/CD28, cluster of differentiation 4, 3, 28; IL-2, interleukin 2; PHA, phytohemagglutinin; PIC, pre-integration complex; IN, integrase; HMG, high mobility group; BAF, barrier to autointegration factor; INI1, integrase interactor 1; LEDGF, lens epithelium-derived growth factor; P/CAF, p300/CBP associated factor; CBP, CREB binding protein; CDK9, cyclin-dependent kinase 9; TPA, 12-O-tetradecanoyl-phorbol-13-acetate; LTR, long terminal repeat TAR, trans-activating response region; SCID, Severe Combined Immune Deficiency; PML, promyelocytic leukemia; PTGS, post-transcriptional gene silencing; RISC, RNA-induced silencing complex; RITS, RNA-induced transcriptional silencing; siRNA, small-inhibitory RNA.

## Competing interests

The author declares that he has no competing interests.
